# Epithelioid Hemangioendothelioma (EHE) Mimicking Mesothelioma: Case Presentation and Review

**DOI:** 10.1155/crom/9459565

**Published:** 2025-02-11

**Authors:** Kabir Grewal, Saivaroon Gajagowni, Elyse Lopez, Kayanaat Grewal, Son Viet Nguyen

**Affiliations:** ^1^Department of Internal Medicine, Baylor College of Medicine, Houston, Texas, USA; ^2^Department of Cancer Medicine, The University of Texas MD Anderson Cancer Center, Houston, Texas, USA; ^3^Department of Medicine, Florida State University College of Medicine, Tallahassee, Florida, USA; ^4^Department of Hospital Medicine, The University of Texas MD Anderson Cancer Center, Houston, Texas, USA

## Abstract

Epithelioid hemangioendothelioma (EHE) is an extremely rare vascular sarcoma that can initially present with a myriad of symptoms anywhere in the body. Imaging findings are often nonspecific, and the pathology tends to have overlap with other malignancies. As a result, it can be quite difficult to suspect and diagnose EHE. We present a case of pulmonary EHE in a 41-year-old female with left pleural thickening and subclavian tumor thrombus who was initially misdiagnosed and treated as mesothelioma. This instance demonstrates the importance of maintaining a broad differential and the utilization of repeat biopsies and next-generation sequencing for questionable diagnoses of atypical malignancies.

## 1. Background

Epithelioid hemangioendothelioma (EHE) is a rare vascular sarcoma with low to moderate differentiation and potential metastatic ability. The incidence of the disease is 0.038/100,000 with an overall prevalence of < 1/1,000,000 [1]. The most commonly affected organs include the liver; bone; and rarely, the lung. There are fewer than 200 cases of primary pulmonary EHE published in the peer-reviewed literature [2]. Presentation can vary from asymptomatic patients with incidental findings on imaging to pleuritic chest pain, dyspnea, and cough [3]. Radiographic findings can include single or multiple lung masses distributed either unilaterally or bilaterally [4]. Disease rarity and nonspecific symptoms and imaging findings make diagnosing EHE difficult.

We present an informative case of pleural EHE that was initially misdiagnosed and treated as mesothelioma. The case highlights the diagnostic difficulty that EHE provides and emphasizes the importance of a broad differential for lung and pleural masses. Immunostaining of biopsy samples showed mixed results, requiring repeat biopsies and staining for official diagnosis.

## 2. Case Presentation

**Figure 1:**
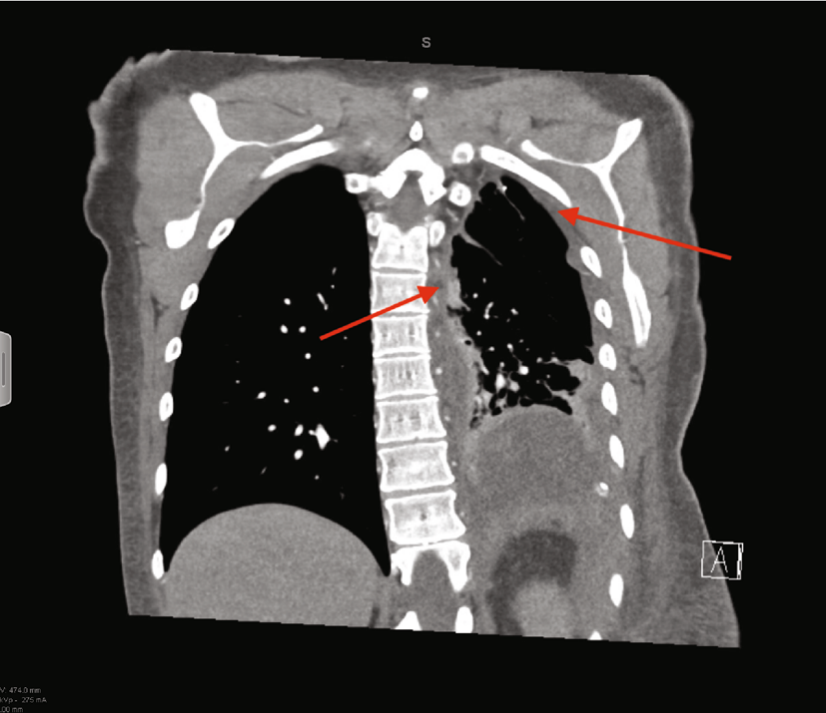
CT of the thorax (coronal). Diffuse nodular thickening of the left pleura with mediastinal lymphadenopathy.

In this case, we focus on a 41-year-old female nonsmoker with no notable medical history, family history of cancer, or known asbestos exposure. She initially presented to her local hospital for a 3-month history of worsening dyspnea, flank pain, and left supraclavicular swelling. Imaging showed a subclavian tumor thrombus; left pleural effusion with diffuse nodular thickening; and mediastinal, supraclavicular, and hilar lymphadenopathy (Figure 1).

At the outside hospital, the patient underwent video-assisted thoracoscopic (VATS) biopsy with subsequent talc pleurodesis. Biopsy from the left supraclavicular lymph node was also obtained. Preliminary pathology results were nonspecific with initial staining grossly positive for WT1 and calretinin. The impression was “poorly differentiated spindle cell neoplasm.” This was presumed to be mesothelioma, and the patient soon underwent Cycle 1 of cisplatin/pemetrexed before being referred to our quaternary care cancer center for further workup and treatment.

## 3. Outcome and Follow-Up

**Figure 2:**
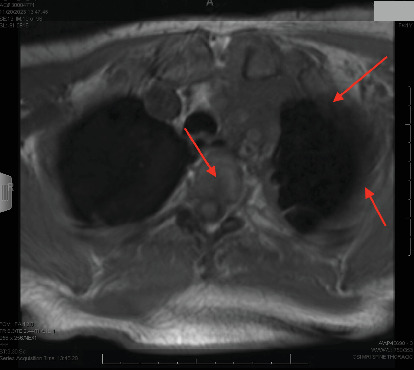
MRI of the thorax (axial). Extensive left-sided pleural thickening and spinal metastases can be seen.

**Figure 3:**
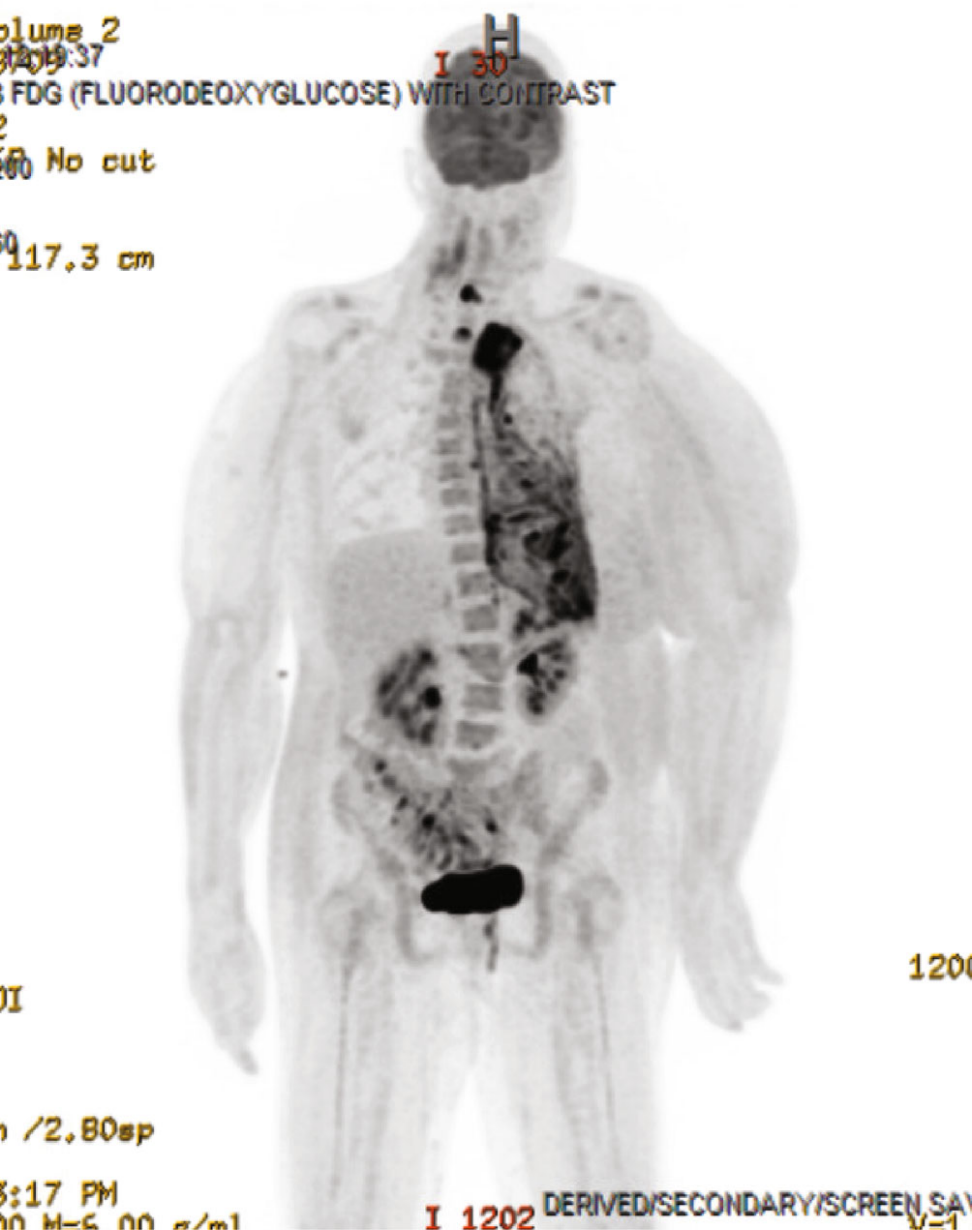
PET-CT (scout). Extensive left lung involvement and activity with diffuse lymphadenopathy.

**Figure 4:**
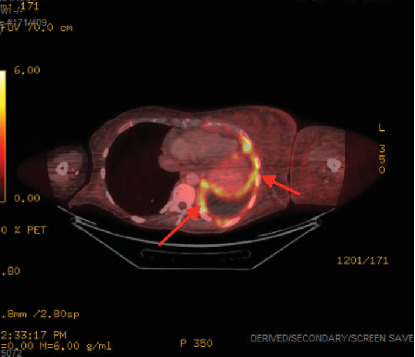
PET-CT (axial). Persistent left lung pleural thickening with increased activity.

**Figure 5:**
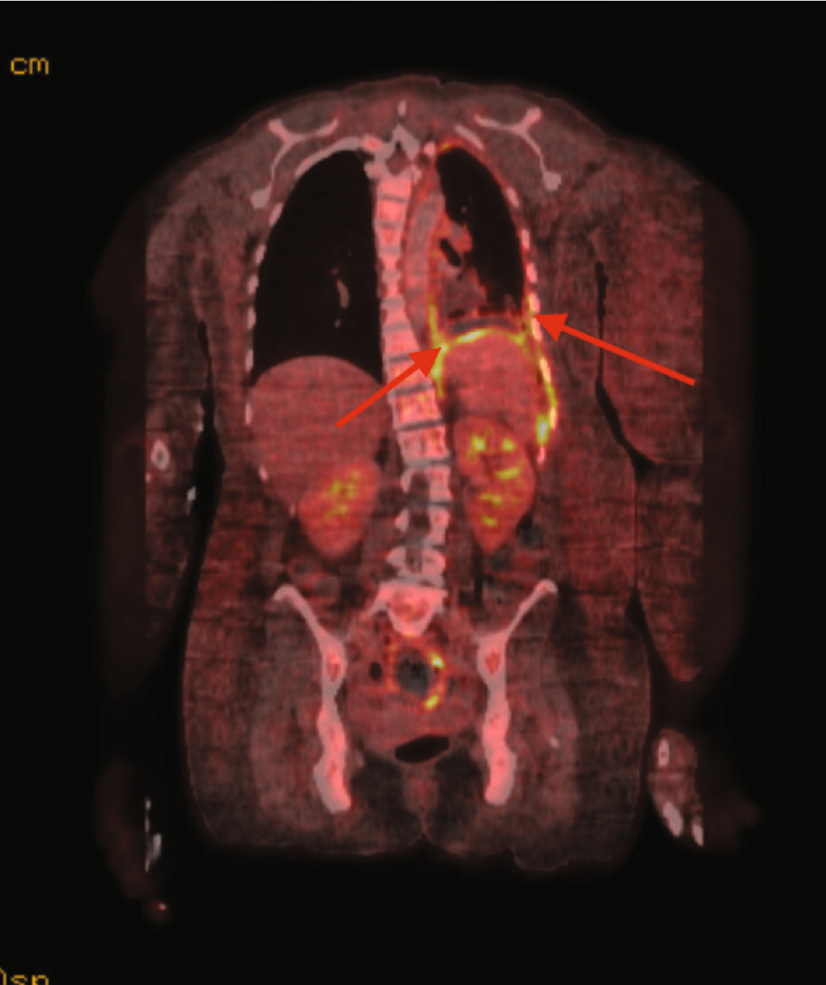
PET-CT (coronal). Diffuse pleural thickening with high activity encapsulating the entire left lung.

The patient presented to our hospital 3 weeks after initial workup and 1 week after initial chemotherapy. She continued to endorse dyspnea, chest tightness, and back pain. MRI showed extensive left-sided pleural disease with osseous metastases to the spine and ribs (Figure 2). PET-CT showed extensive left-sided pleural thickening with soft tissue extension into the neck and involvement of the left brachiocephalic and jugular veins (Figures 3, 4, and 5). A CT-guided percutaneous left pleural biopsy (core + FNA) was performed.

Immunohistochemical studies were grossly negative for cytokeratin, calretinin, and WT1, which was inconsistent with the original mesothelioma diagnosis. Repeat pathology staining was positive for ERG, CD31, and nuclear CAMTA-1. After extension discussion between pathology and the sarcoma team, a diagnosis of EHE was established. The detection of a WWTR1:CAMTA-1 fusion alteration on gene sequencing helped confirm EHE.

The patient was started on a chemotherapy regimen of gemcitabine and docetaxel for a goal of 10 total cycles of 21 days each. After six cycles, surveillance imaging shows no further disease progression. Due to pancytopenia, Cycle 7 was delayed and the patient was put on a treatment break with plans to resume chemo in the future pending follow-up.

## 4. Discussion

We present a case of a 41-year-old female with EHE involving the pleura. Literature review shows 58 published cases of pleural EHE (Table 1). Sixty-four percent of the patients were male, and the average age at diagnosis was 50. Thirty-three cases (57%) are confirmed to be dead, with 22 of the 33 dying within 12 months of diagnosis. Pleural involvement is associated with poor prognosis [44]. Treatment modalities varied, with 11 (19%) receiving only surgical interventions, 15 (26%) receiving only chemotherapy, 10 (17%) receiving a combination of surgery/chemotherapy/radiation, and 22 (38%) not receiving any form of treatment or not having data available.

Diagnosis of pulmonary EHE is complicated by a lack of specific symptoms. The most common initial presentation among published cases is pleuritic chest pain (43%), with other common symptoms including dyspnea and cough (Table 1). Imaging findings are nonspecific and often show pleural thickening with effusions. As in our case, EHE can often be misdiagnosed as mesothelioma [15]. This emphasizes the importance of maintaining a broad differential for pleural thickening including inflammation, infection, mesothelioma, and EHE.

Biopsy remains the gold standard for diagnosing and differentiating between EHE and mesothelioma. EHE classically stains positive for endothelial and vascular markers including CD31, CD34, and ERG [1]. More recently, positive immunohistochemical staining for nuclear CAMTA-1 is found to be specific for EHE [41, 45]. On the other hand, mesothelioma classically stains positive for calretinin, CK5 or CK5/6, WT1, and D2-40 [41].

Preliminary biopsy results for our patient were positive for WT1, calretinin, and D2-40, leading to the initial diagnosis and treatment of mesothelioma. However, repeat biopsy showed immunostaining positive for ERG, CD31, and nuclear CAMTA-1 and negative for WT1, calretinin, and CK5/6, consistent with EHE. This diagnosis was only confirmed after next-generation sequencing showed a WWTR1:CAMTA1 fusion alteration, a classic finding in EHE [45]. The mixed results in this case showcase that biopsies may not be definitive, especially when samples show such conflicting results. Next-generation sequencing can be utilized to differentiate between mimicking diseases on a genomic level.

Due to the rarity of EHE, treatment for the condition varies based on disease site. Early cases of EHE were treated with surgical excision, although often unsuccessfully. More recently, the condition has been treated with chemotherapy, typically carboplatin/etoposide. Due to the rarity and complex nature of EHE, there has been a push to establish global standards of treatment [1]. It is recommended for EHE to be managed at dedicated sarcoma centers. The preferred treatment for unifocal EHE, defined as a localized tumor, is surgery, with an expected cure rate of 70%–80% for negative margins. Other procedures such as pleural stripping or pneumonectomy should be considered individually in cases of pulmonary EHE [44]. Radiation is recommended as an adjunct to surgery, not alone. If there is serosal involvement or metastatic disease, patients are candidates for systemic therapy, although a standard regimen has not been established. Retrospective data suggests that the highest clinical activity is seen with mTOR inhibitors with a progression-free survival (PFS) and overall survival in the range of 1 year and 2 years, respectively, and ∼10% of patients having even longer PFS [1].

We present a rare case of EHE involving the pleura that was misdiagnosed as mesothelioma. This case emphasizes the utility of repeat biopsies to confirm results when the diagnosis is unclear or when the clinical presentation is atypical for the suspected disease (i.e., mesothelioma in a young patient without asbestos exposure). Next-generation sequencing can be useful to confirm disease on a genomic level. Specialty cancer centers can be quite beneficial in establishing and treating ultrarare malignancies like EHE. Further research into establishing localized and systemic treatment guidelines for EHE are needed.

## Figures and Tables

**Table 1:** Literature review of existing cases of pulmonary EHE. Pubmed was utilized using the terms “pulmonary epithelioid hemangioendothelioma,” “epithelioid hemangioendothelioma,” and “EHE.” All articles were in peer-reviewed journals. Cases are ordered chronologically, and initial patient presentation, treatment, and current status are summarized.

**Study**	**Patient demographics**	**Initial presentation**	**Treatment**	**Survival (months)**
Stout, 1943 [5]	61 M	Chest pain, night sweats, hemoptysis	None	8
Yousem and Hochholzer, 1987 [6]	34 M	Dyspnea	None	3
Bevelaqua, Valensi, and Hulnick, 1988 [7]	22 M	Fever	Lobectomy	NR
Lin et al., 1996 [8]	50 M	Pleuritic pain	Pleurectomy, pneumonectomy	Died, unclear time frame
	34 F	Not available	Not available	Died, unclear time frame
	42 M	Pleural effusion	Pleurectomy	Not available
	56 M	Thoracic outlet syndrome	Pleurectomy	Died, unclear time frame
	58 M	Pleural effusion	Pleurectomy	Died, unclear time frame
	36 M	Pleural effusion, pericardial effusion	Pleurectomy	Alive at time of study
	51 M	Pleural effusion	Pleurectomy	Died, unclear time frame
Pinet et al., 1999 [9]	50 F	Right pleural effusion	Carboplatin, etoposide	Complete remission at 18 months
Crotty et al., 2000 (*n* = 4) [10]	55–71 M	Chest pain, dyspnea, cough, fever, weight loss	N/A	3/4 died within 10 months; the 4th patient was lost to follow-up
Cronin and Arenberg, 2004 [11]	35 F	Dyspnea, cough	Interferon alpha, gemcitabine, docetaxel	9
Vitorio, 2004 [12]	61 M	Chest pain	Cisplatin/etoposide	3
Al-Shraim et al., 2005 [13]	51 M	Dry cough, dyspnea	Interferon alpha	NR
Saqi et al., 2007 [14]	37 M	Dyspnea, pleuritic chest pain	Pleural decortication, carboplatin/etoposide, bevacizumab	NR
Bahrami, Allen, and Cagle, 2008 [15]	37 F	Back pain	Radiotherapy, cisplatin/etopside	11
Lee et al., 2008 [16]	31 F	Back pain	Adriamycin, transitioned to mesna–doxorubicin–ifosfamide–dacarbazine	10
André et al., 2010 [17]	65 F	Right-sided chest pain	Carboplatin, etoposide	6
Liu, Shiau, and Nonaka, 2010 [18]	80 M	Dyspnea	Chest tube placement, chemotherapy	6
Bocchino et al., 2010 [19]	58 F	Dyspnea, cough, chest pain	None	3
Lazarus et al., 2011 [20]	42 M	Cough, dyspnea, chest pain	Paclitaxel, bevacizumab	8
	42 M	Fever, cough	Carboplatin/etoposide, bevacizumab	6
Kim, Lele, and Lackner, 2011 [21]	46 F	Right-sided chest pain, cough	Pleurectomy, carboplatin/etoposide	Disease progression but unclear on status
Chou, Hsieh, and Wu, 2011 [22]	54 M	Cough	Wedge resection	Disease-free at 16 months
	42 M	Chest pain, cough	Pleurectomy, radiotherapy, chemotherapy	NR
	27 M	Cough, chest tenderness, hoarseness	Pleurectomy, radiotherapy, chemotherapy	18
Márquez-Medina et al., 2011 [23]	85 M	Chest pain, weight loss	None	7
Bansal et al., 2012 [24]	51 F	Chest pain	Doxorubicin	4
Yu et al., 2013 [25]	39 F	Dyspnea	Surgical resection, carboplatin/etoposide	Alive at time of study
Ha et al., 2014 [26]	71 M	Cough, dyspnea	Not reported	Not reported
Salijevska et al., 2015 [27]	36 F	Right-sided chest pain	Pleurodesis, paclitaxel	6
Wethasinghe et al., 2015 [28]	41 M	Right-sided chest pain, weight loss	Radiotherapy	Alive at time of study
Apolinário et al., 2016 [29]	47 M	Chest pain, dyspnea	Doxorubicin	6
Kanemura et al., 2016 [30]	31 F	Left back pain	Carboplatin, pemetrexed, and bevacizumab	Alive at time of study
Fan et al., 2016 [31]	68 F	Back pain	None	11
Albano et al., 2017 [32]	76 F	Dyspnea, coughing, and chest pain	N/A	6
Fjaellegaard et al., 2020 [33]	71 M	Dyspnea, cough	N/A	Died before treatment initiation
Takenaka et al., 2020 [34]	62 M	Chest pain, dyspnea	Extrapleural pneumonectomy, pazopanib	3.5
Abd El Hafez, 2021 [35]	41 M	Dyspnea, hemoptysis	N/A	Unclear
Askari et al., 2021 [36]	40 M	Right-sided chest pain with dyspnea	Adriamycin and ifosfamide	Alive at time of study
Lavacchi et al., 2021 [37]	53 M	Left chest wall pain	Extrapleural pneumonectomy	Palliative surgery
Al-Baali, Al-Mahrouqi, and Al-Sawafi, 2022 [38]	65 F	Pleuritic chest pain, cough, and dyspnea	Paclitaxel	Alive at time of study
Rezvani et al., 2022 [39]	68 M	Pleuritic chest pain and weight loss	Pazopanib, paclitaxel	Lost to follow-up
Pathak and Walker, 2023 [40]	73 F	Dyspnea, pleural effusion	Decortication	Alive at time of study
Weissferdt and Moran, 2023 [41]	35 M	Dyspnea, chest pain	Not available	8
	41 F	Cough, dyspnea	Not available	14
	37 M	Dyspnea	Not available	30
	65 F	Cough, chest pain	Not available	16
	56 F	Chest pain	Not available	22
	48 M	Chest pain	Not available	26
	54 M	Chest pain	Not available	Not available
	43 M	Dyspnea, cough	Not available	Not available
Nguyen et al., 2023 [42]	40 F	Dyspnea, cough, left-sided chest pain	Radiation	Alive at time of study
Bashir Mohamed et al., 2023 [43]	51 F	Cough	Wedge resection	Alive at time of study
Present case	41 F	Chest pain	Pleurodesis, cisplatin/etoposide/gemcitabine/docetaxel	Alive at time of study

## Data Availability

The data that support the findings of this study are available on request from the corresponding author. The data are not publicly available due to privacy or ethical restrictions.
